# Blue Mussel-Derived Peptides PIISVYWK and FSVVPSPK Trigger Wnt/β-Catenin Signaling-Mediated Osteogenesis in Human Bone Marrow Mesenchymal Stem Cells

**DOI:** 10.3390/md18100510

**Published:** 2020-10-09

**Authors:** Yunok Oh, Chang-Bum Ahn, Jae-Young Je

**Affiliations:** 1 Institute of Marine Life Sciences, Pukyong National University, Busan 48613, Korea; si565@daum.net; 2 Division of Food and Nutrition, Chonnam National University, Gwangju 61186, Korea; a321@jnu.ac.kr; 3 Department of Marine-Bio Convergence Science, Pukyong National University, Busan 48547, Korea

**Keywords:** osteogenic peptides, osteoblast differentiation, Wnt/β-catenin signaling, human mesenchymal stem cells

## Abstract

Marine-derived bioactive peptides have shown potential bone health promoting effects. Although various marine-derived bioactive peptides have potential nutraceutical or pharmaceutical properties, only a few of them are commercially available. This study presented an osteogenic mechanism of blue mussel-derived peptides PIISVYWK and FSVVPSPK as potential bone health promoting agents in human bone marrow-derived mesenchymal stem cells (hBMMSCs). Alkaline phosphatase (ALP) activity and mineralization were stimulated using PIISVYWK and FSVVPSPK as early and late markers of osteogenesis in a concentration-dependent manner. Western blot and RT-qPCR results revealed that PIISVYWK and FSVVPSPK increased osteoblast differentiation of hBMMSCs by activating canonical Wnt/β-catenin signaling-related proteins and mRNAs. Immunofluorescence images confirmed nuclear translocation of β-catenin in osteogenic differentiation. Treatment with the pharmacological inhibitor DKK-1 blocked PIISVYWK- and FSVVPSPK-induced ALP activity and mineralization, as well as mRNA expression of the canonical Wnt/β-catenin signaling pathway in hBMMSC differentiation into osteoblasts. These findings suggested that PIISVYWK and FSVVPSPK promoted the canonical Wnt/β-catenin signaling pathway in osteogenesis of hBMMSCs. Blue mussel-derived PIISVYWK and FSVVPSPK might help develop peptide-based therapeutic agents for bone-related diseases.

## 1. Introduction

Osteoporosis is one of the most common diseases caused by an imbalance between bone resorption by osteoclasts and bone formation by osteoblasts. The aging population is more prone to osteoporosis as a result of bone resorption rates being higher than bone formation rates [1]. Antiresorptive therapies are currently used to treat osteoporosis. The main classes of these agents are bisphosphonates, calcium, and estrogen, and they help maintain bone mass by inhibiting osteoclasts [2]. However, there are certain risks associated with these treatments, such as necrosis of the jaw [3]. A number of bone-forming agents, derived from natural products, have been developed to treat osteoporosis by enhancing mesenchymal stem cell (MSC) differentiation into osteoblasts [4,5,6,7]. MSCs can differentiate into osteogenic, chondrogenic, and adipogenic lineage cells [8]. Several signaling pathways, such as Wnt/β-catenin, bone morphogenetic proteins (BMPs), mitogen-activated protein kinase (MAPK), and notch, are involved in the regulation of MSC differentiation into osteoblasts [9,10]. Many studies have revealed that Wnt signaling plays an important role in bone repair [11,12]. Accumulating evidence from these studies has shown that the use of MSCs is a promising strategy in treating bone-related diseases, such as fractures [11]. Consequently, it may be helpful to develop substances that are capable of regulating MSC differentiation into osteoblasts for bone-related disease treatment.

Marine organisms are recognized as a potential source of bioactive compounds. Due to a unique environment, bioactive compounds in marine organisms show potential health benefits, including anticancer, antioxidant, anti-inflammatory, antimicrobial, and antihypertensive activities [13,14,15,16,17,18,19]. Recently, marine organism-derived bioactive peptides (BAPs) have gained much attention due to their broad spectra of bioactivities. BAPs can be obtained by proteolytic cleavage with specific proteases and identified as promising nutraceuticals or pharmaceuticals [20]. In addition, studies show that BAPs can regulate MSC differentiation into osteoblasts by activating specific signaling pathways, such as Wnt signaling [21,22,23,24]. Recently, our group developed BAPs that can regulate MSC differentiation into osteoblasts and adipocytes [25,26,27,28]. These studies show that those BAPs upregulate BMPs or MAPK-dependent BMP signaling pathways in the regulation of MSC differentiation into osteoblasts. However, Wnt/β-catenin signaling-mediated osteoblast differentiation by those BAPs is still unexplored. Previously, we identified two antioxidant peptides (PIISVYWK and FSVVPSPK) with a hepatoprotective effect from the blue mussel [29]. Therefore, as part of our ongoing investigation on BAP functions, we investigated the potential role of PIISVYWK and FSVVPSPK in human bone marrow-derived MSC (hBMMSC) differentiation into osteoblasts through the Wnt/β-catenin signaling pathway.

## 2. Results

### 2.1. Blue Mussel-Derived Peptides PIISVYWK and FSVVPSPK Increase Osteoblast Differentiation in Human Bone Marrow-Derived Mesenchymal Stem Cells (hBMMSCs)

**Figure 1:**
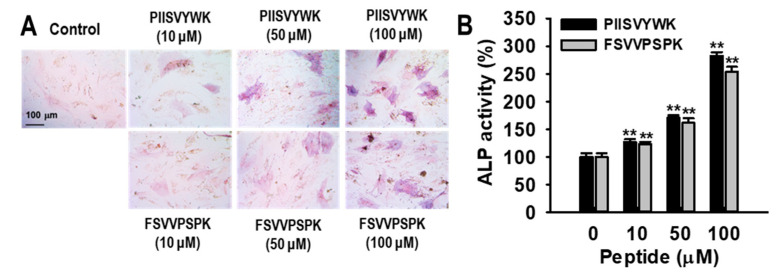
Effect of PIISVYWK and FSVVPSPK on alkaline phosphatase (ALP) activity in osteoblast differentiation of human bone marrow-derived mesenchymal stem cells (hBMMSCs). (**A**) Representative image of ALP staining and (**B**) quantification of ALP activity. hBMMSCs were treated with peptides in an osteogenic medium for 7 days. Values are presented as means ± SD of four independent determinations (n = 4). ** *p* < 0.01 vs. non-treatment group.

**Figure 2:**
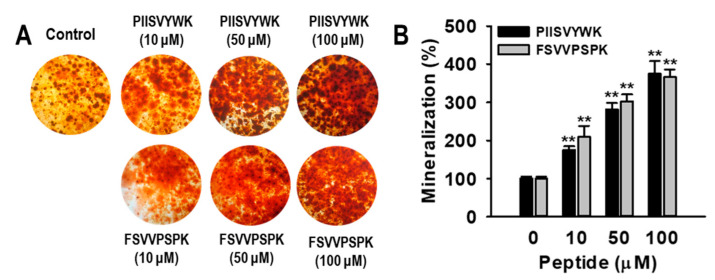
PIISVYWK and FSVVPSPK promoted mineralization in osteoblast differentiation of hBMMSCs. (**A**) Representative images of Alizarin Red S (ARS) and (**B**) quantification of mineralization. hBMMSCs were treated with peptides in an osteogenic medium for 21 days. Values are presented as means ± SD of four independent determinations (n = 4). ** *p* < 0.01 vs. non-treatment group.

The effects of PIISVYWK and FSVVPSPK on hBMMSC viability were determined using a 3-[4,5 -dimethylthiazol-2-yl]-2,5-diphenyltetrazolium bromide (MTT) assay. No effects were observed in concentrations up to 100 μM (data not shown). To investigate the effects of PIISVYWK and FSVVPSPK on hBMMSC differentiation into osteoblasts, hBMMSCs were treated with osteogenic medium (OM) in the presence or absence of PIISVYWK and FSVVPSPK (0~100 μM). After incubation for 7 days, ALP activity as an early stage marker in osteoblast differentiation was first determined. As depicted in Figure 1, ALP activity in the presence of PIISVYWK and FSVVPSPK was significantly increased compared to that of the cells without the peptides, and the stimulation was dose-dependent. Staining assay results showed that PIISVYWK and FSVVPSPK increased the intensity of ALP staining. Next, mineralization as a late-stage marker in osteoblast differentiation was determined using Alizarin Red S (ARS) staining. It was found that PIISVYWK and FSVVPSPK increased mineralization. Quantification of ARS staining showed 375 ± 18% and 365 ± 20% increments compared to that of the cells without PIISVYWK and FSVVPSPK (Figure 2).

### 2.2. PIISVYWK and FSVVPSPK Activates Wnt/Β-Catenin Signaling

**Figure 3:**
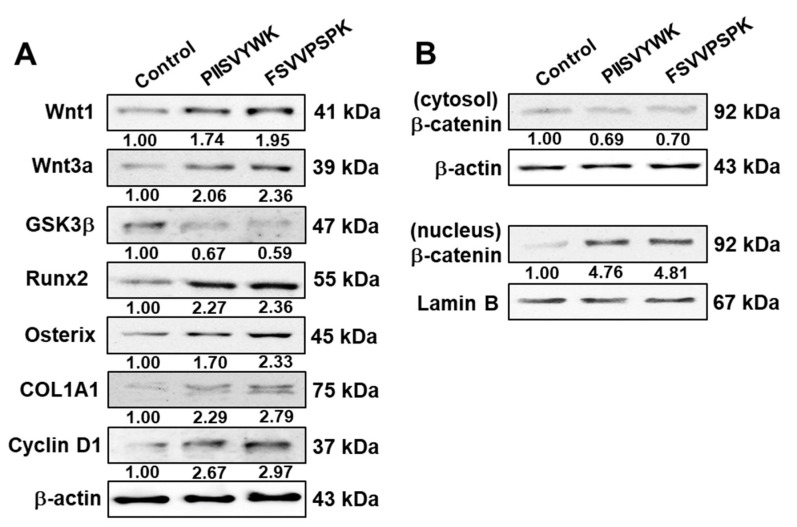
Effect of PIISVYWK and FSVVPSPK on the Wnt/β-catenin signaling pathway in osteoblast differentiation of hBMMSCs. Cells were treated with PIISVYWK and FSVVPSPK (100 μM) in an osteogenic medium for 7 days (**A**) and 4 days (**B**). Protein expression was assessed using Western blot analysis. All experiments were done in triplicate.

**Figure 4:**
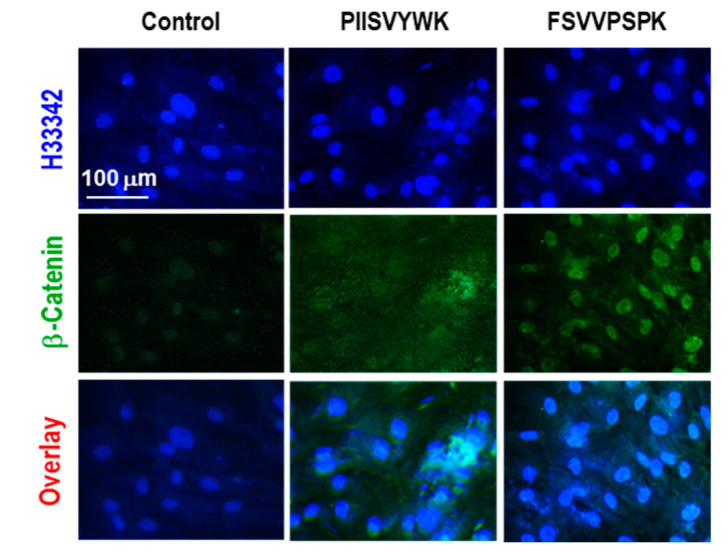
PIISVYWK and FSVVPSPK promoted nuclear translocation of β-catenin in osteoblast differentiation of hBMMSCs. Cells were cultured in an osteogenic medium with PIISVYWK and FSVVPSPK (100 μM) for 4 days. β-catenin was immunostained with a β-catenin-specific monoclonal antibody, followed by an Alexa Flour 488-conjugated secondary antibody. Cells were then stained with H33342 (blue). All experiments were done in triplicate.

**Figure 5:**
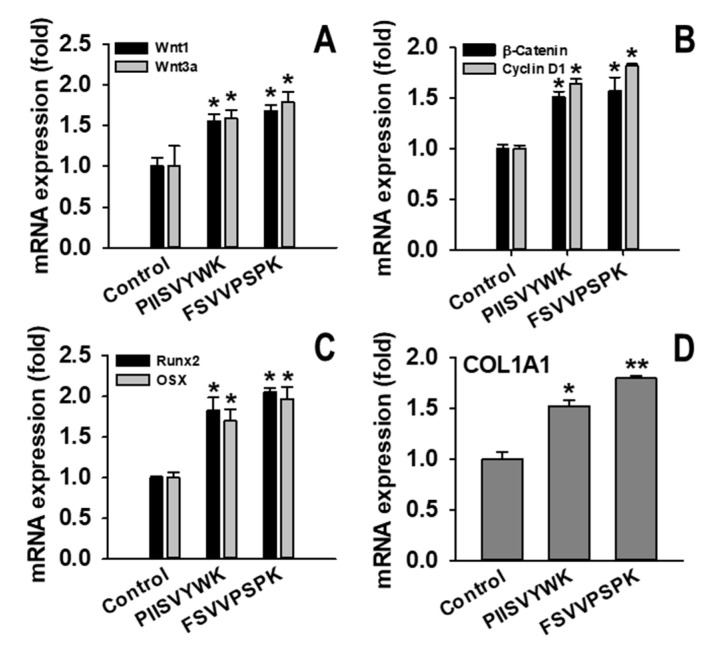
Effect of PIISVYWK and FSVVPSPK on mRNA expression of Wnt/β-catenin signaling in osteoblast differentiation of hBMMSCs. (**A**) Wnt1 and Wnt3a, (**B**) β-catenin and cyclin D1, (**C**) Runx2 and OSX, and (**D**) COL1A1. Cells were cultured in osteogenic medium with PIISVYWK and FSVVPSPK (100 μM) for 7 or 4 days (only for β-catenin). mRNA expression was assessed using RT-qPCR. Values are presented as means ± SD of three independent determinations (n = 3). * *p* < 0.05, ** *p* < 0.01 vs. control group (without peptide treatment).

The canonical Wnt/β-catenin signaling pathway is known to be involved in osteoblast differentiation of MSCs [30]. To understand the underlying molecular mechanism of hBMMSC differentiation into osteoblasts with PIISVYWK and FSVVPSPK, activation of the Wnt/β-catenin signaling pathway was investigated via Western blotting, immunofluorescence, and RT-qPCR. First, the effects of PIISVYWK and FSVVPSPK on protein expression of Wnt ligands, GSK3β, Runx2, OSX, COL1A1, cyclin D1, as well as β-catenin in the cytosolic and nuclear fractions, were examined. As shown in Figure 3A, hBMMSCs treated with the peptides showed upregulation of Wnt1, Wnt3a, Runx2, OSX, COL1A1, and cyclin D1, and downregulation of GSK3β. Protein expression of β-catenin in the cytosolic and nucleus fractions showed downregulation of cytosolic β-catenin and upregulation of nuclear β-catenin, indicating translocation of β-catenin into the nucleus (Figure 3B). An immunofluorescence assay was performed after treating the peptides to confirm nuclear translocation of β-catenin. As seen in Figure 4, immunofluorescence analysis showed that PIISVYWK and FSVVPSPK rapidly stimulated nuclear translocation of β-catenin in hBMMSCs. mRNA expression of Wnt1, Wnt3a, Runx2, OSX, COL1A1, cyclin D1, and β-catenin was significantly increased in the presence of PIISVYWK and FSVVPSPK (Figure 5).

### 2.3. Dickkopf-1 (DKK-1) Reversed PIISVYWK and FSVVPSPK-Enhanced Osteoblast Differentiation

**Figure 6:**
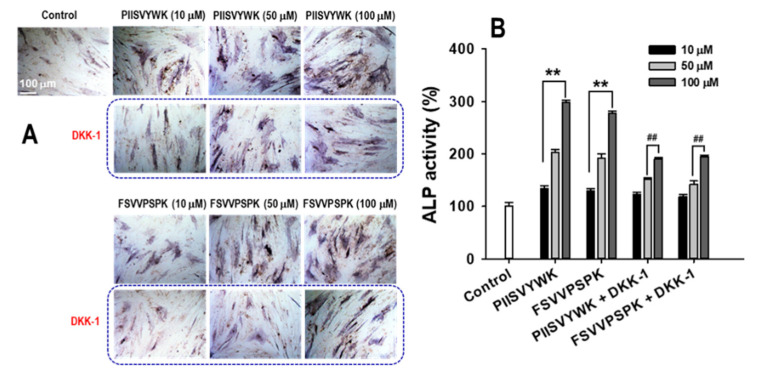
DKK-1 treatment abolished PIISVYWK- and FSVVPSPK-mediated ALP activity in osteoblast differentiation of hBMMSCs. (**A**) Representative images of ALP staining and (**B**) quantification of ALP activity. Cells were exposed to DKK-1 (100 ng/mL) for 1 h, followed by the addition of PIISVYWK and FSVVPSPK, and incubated for 7 days. Values are presented as means ± SD of four independent determinations (n = 4). ** *p* < 0.01 vs. control group (without peptide treatment), ^##^ *p* < 0.01 vs. PIISVYWK and FSVVPSPK.

To test whether PIISVYWK and FSVVPSPK induced osteoblast differentiation by activating the Wnt/β-catenin signaling pathway, cells were treated with DKK-1 (100 ng/mL), an antagonist of Wnt signaling, for 1 h prior to exposing the peptides. On day 7, ALP activity with and without DKK-1 in the presence of the peptides was determined. As shown in Figure 6, the peptide-treated cells showed higher ALP activity than the cells without the peptides, whereas the cells pretreated with DKK-1 showed lower ALP activity than the cells treated with the peptides. ALP staining results were consistent with the ALP activity results.

**Figure 7:**
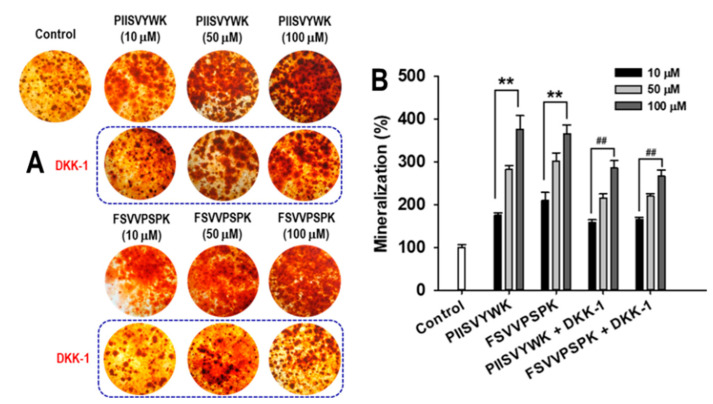
DKK-1 treatment abolished PIISVYWK- and FSVVPSPK-mediated mineralization in osteoblast differentiation of hBMMSCs. (**A**) Representative images of ARS and (**B**) quantification of mineralization. Cells were exposed to DKK-1 (100 ng/mL) for 1 h, followed by addition of PIISVYWK and FSVVPSPK, and incubated for 21 days. Values are presented as means ± SD of four independent determinations (n = 4). ** *p* < 0.01 vs. control group (without peptide treatment), ^##^ *p* < 0.01 vs. PIISVYWK and FSVVPSPK.

**Figure 8:**
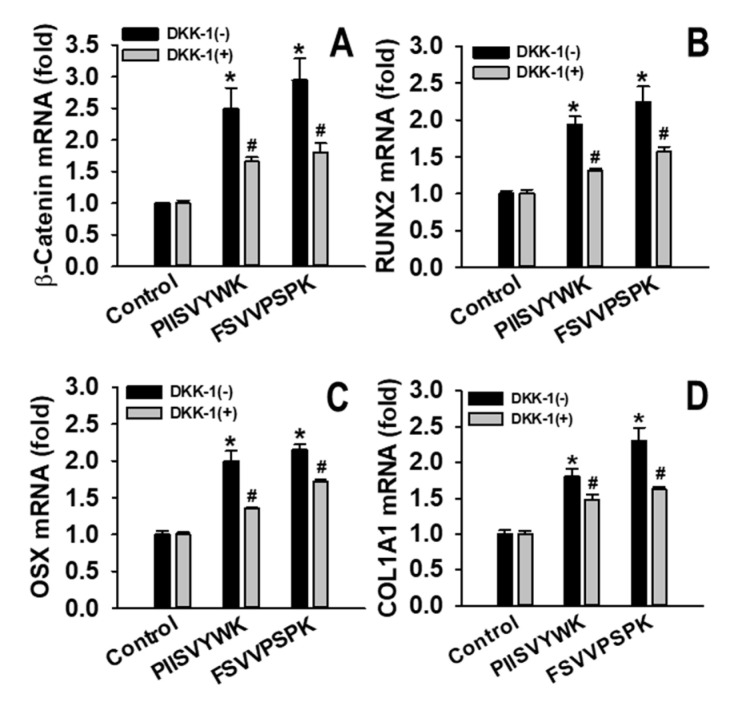
Effect of DKK-1 on mRNA expression of (**A**) β-catenin, (**B**) Runx2, (**C**) OSX, and (**D**) COL1A1 in PIISVYWK- and FSVVPSPK-mediated osteoblast differentiation in hBMMSCs. Cells were exposed to DKK-1 (100 ng/mL) for 1 h, followed by addition of PIISVYWK and FSVVPSPK, and incubated for 7 or 4 days (only for β-catenin). Values are presented as means ± SD of three independent determinations (n = 3). * *p* < 0.01 vs. control group (without peptide treatment), ^#^ *p* < 0.01 vs. PIISVYWK and FSVVPSPK without DKK-1.

The effect of DKK-1 on mineralization was also examined. On day 21, the degree of mineralization with and without DKK-1 in the presence of the peptides was determined (Figure 7). Results showed that DKK-1 pretreatment significantly decreased the degree of mineralization compared to that of the cells without DKK-1. To further examine the involvement of DKK-1 pretreatment in Wnt/β-catenin signaling, mRNA levels of β-catenin, Runx2, OSX, and COL1A1 were examined. As shown in Figure 8, DDK-1 pretreatment attenuated PIISVYWK- and FSVVPSPK-mediated mRNA expression of Wnt/β-catenin signaling. These findings suggested that the Wnt/β-catenin signaling pathway was required for PIISVYWK- and FSVVPSPK-induced hBMMSC differentiation into osteoblasts.

## 3. Discussion

Bone formation is a complicated process that includes osteoblast proliferation, differentiation and, ultimately, formation of mineralized bone matrix comprising COL1A1 fibers. ALP is a good indicator of bone formation and is a major regulator of phosphate supplementation in bone mineralization [31]. ALP is a relative early-stage marker of osteoblast differentiation, while mineralization is considered a late-stage marker of osteoblast differentiation. In this study, we demonstrated that PIISVYWK and FSVVPSPK promoted ALP activity and mineralization of hBMMSCs and increased protein and mRNA expressions of COL1A1. These results indicated that osteoblast differentiation and mineralized bone matrix formation were stimulated by PIISVYWK and FSVVPSPK.

Considerable studies have shown that the Wnt signaling pathway is involved in regulation of bone formation and MSC differentiation into osteoblasts [6,7,32]. Wnt signaling regulates cell fate and commits cells to the osteoblast lineage, differentiation into mature osteoblasts, and mineralization [30]. Inactivation of Wnt signaling leads to MSC differentiation into chondrocytes or adipocytes [33]. Two signaling pathways by Wnt ligands are involved in osteoblast differentiation. The β-catenin-independent non-canonical pathway is mediated by the Wnt5a ligand, whereas the β-catenin-dependent canonical pathway is mediated by Wnt1 and Wnt3a. In the canonical Wnt/β-catenin signaling pathway, Wnt ligands bind to the receptor complex of Frizzled and LRP5/6. This binding, in turn, inhibits GSK3β phosphorylation of its substrate β-catenin. Thus, Wnt ligands inhibit proteolysis of β-catenin through GSK3β inhibition, leading to cytosolic accumulation and translocation of β-catenin into the nucleus in the target cells [30,34]. Nuclear β-catenin triggers the expression of Wnt target genes, including Runx2 [35]. Runx2 is the master transcription factor essential for osteoblast differentiation and bond formation by upregulation of OSX, COL1A1, and osteocalcin [7,35]. Therefore, activation of Wnt signaling by specific agents is an important strategy in preventing bone-related diseases. Our observation showed that PIISVYWK- and FSVVPSPK-treated hBMMSCs enhanced protein expressions of Wnt1 and Wnt3a and downregulated the protein expression of GSK3β, ultimately stimulating nuclear translocation of β-catenin, as confirmed by Western blot and immunostaining analysis. Eventually, Runx2 and OSX, as well as cyclin D1, the target gene of β-catenin, were upregulated by PIISVYWK and FSVVPSPK treatment in hBMMSCs. We also showed upregulated mRNA levels of these proteins. These results suggested that PIISVYWK and FSVVPSPK promoted hBMMSC differentiation into osteoblasts through the canonical Wnt/β-catenin signaling pathway.

To investigate the role of Wnt/β-catenin in hBMMSC differentiation with PIISVYWK and FSVVPSPK, we used DKK-1, an antagonist of the canonical Wnt/β-catenin signaling. DKK-1 can bind to LRP5/6, thereby preventing the interaction between Wnt ligands and their receptors for signal transduction. Overexpression of DKK-1 downregulated endogenous β-catenin and ALP activity in osteoblasts and, ultimately, DDK-1 inhibited bone formation [36]. In this study, we showed that PIISVYWK- and FSVVPSPK-mediated osteogenic effects, including ALP activity and mineralization, were abolished by DKK-1. In addition, pretreatment with DKK-1 decreased mRNA expressions of β-catenin, Runx2, OSX, and COL1A1. These results suggested that PIISVYWK and FSVVPSPK promoted hBMMSCs differentiation into osteoblasts through the canonical Wnt/β-catenin signaling pathway.

## 4. Materials and Methods

### 4.1. Peptide Synthesis

Blue mussel-derived peptides (PIISVYWK and FSVVPSPK) were chemically synthesized and supplied by Peptron Inc. (Daejeon, Korea).

### 4.2. Osteogenic Differentiation and Treatment of Peptides

Human bone marrow-derived mesenchymal stem cells (hBMMSCs, PCS-500-012) were obtained from the American Type Culture Collection (ATCC, Manassan, VA, USA). hBMMSCs were cultured in Dulbecco’s Modified Eagle’s Medium (DMEM, HyClone, Logan, UT, USA) supplemented with 10% fetal bovine serum and 1% penicillin/streptomycin (Gibco, Waltham, MA, USA) in a humidified incubator with 5% CO_2_ at 37 °C. When the cells were 80–90% confluent, osteoblastic differentiation was induced by changing to an osteogenic medium (OM, DMEM supplemented with 10^−7^ M dexamethasone, 50 µg/mL ascorbic acid, and 10 mM β-glycerolphosphate). To test the effects of PIISVYWK and FSVVPSPK on osteoblastic differentiation, cells were treated with OM in the presence of peptides. All media were changed every 2 days.

### 4.3. MTT Assay

After treatment, MTT solution (0.5 mg/mL) was added to the cells, incubated for 4 h for the metabolism of MTT to formazan crystals, and insoluble formazan crystals were dissolved in dimethyl sulfoxide (DMSO). Absorbance was measured at 540 nm (GENios microplate reader, GENios, TECAN, Männedorf, Switzerland).

### 4.4. ALP Activity

ALP activity was measured using staining and spectrophotometry. At day 7 of culturing, the cells were washed with PBS and then fixed with 10% formalin for 5 min at room temperature. Then, the cells were rinsed with PBS three times and stained with NBT/BCIP substrate solution (Roche Diagnostics, Mannheim, Germany) in incubation for 10 min at 37 °C. The cells were washed with deionized water after aspirating the staining solution.

To measure ALP activity, at day 7 of culturing the cells were washed and lysed with sodium carbonate buffer (25 mM, pH 10) supplemented with 1.0% triton X-100. ALP activity in the supernatant of the lysate was measured according to our previous method [25].

### 4.5. Mineralization Assay

At day 21 of culturing, the cells were rinsed with deionized water four times and fixed with 70% ethanol for 1 h at 4 °C. Finally, the cells were stained with 2% ARS (pH 4.2) for 15 min with gentle agitation at room temperature, followed by washing in deionized water. The calcium deposit level was observed using a light microscope. The deposit was dissolved in 10% cetylpyridium chloride in 10 mM sodium phosphate buffer (pH 7.0) for 15 min, and absorbance was measured at 562 nm [25].

### 4.6. Western Blot Analysis

After 4 or 7 days of culturing, the cells were washed with ice-cold PBS and lysed with a RIPA buffer containing a protease inhibitor (Roche Applied Science, Indianapolis, IN, USA). For β-catenin analysis, cytosolic and nuclear fractions were prepared using a nuclear extraction kit (Abcam, Cambridge, MA, USA) according to the manufacturer’s instructions. Protein concentration was determined using a Pierce^TM^ BCA Protein Assay Kit (Pierce Biotechnology, Rockford, IL, USA). Protein was separated on SDS-PAGE and transferred to a nitrocellulose membrane, followed by blocking with 5% skim milk in tris-buffered saline containing 0.1% Tween 20 (TBS-T) for 1 h at room temperature. The membrane was incubated overnight at 4 °C with primary antibodies as follows: β-Actin (Cat. No. sc-47778, 1:500; Santa Cruz Biotechnology, Santa Cruz, CA, USA) and lamin B (Cat. No. sc-374015, 1:500; Santa Cruz Biotechnology), Wnt1 (Cat. No. sc-514531, 1:200; Santa Cruz Biotechnology), Wnt3a (Cat. No. sc-136163, 1:200; Santa Cruz Biotechnology), RUNX2 (Cat. No. sc-390715, 1:200; Santa Cruz Biotechnology), Osterix (Cat. No. sc-393060, 1:200; Santa Cruz Biotechnology), GSK3β (Cat. No. sc-71186, 1:200; Santa Cruz Biotechnology), COL1A1 (Cat. No. sc-59772, 1:200; Santa Cruz Biotechnology), cyclin D1 (Cat. No. sc-8396, 1:200; Santa Cruz Biotechnology), and β-catenin (Cat. No. sc-7963, 1:200; Santa Cruz Biotechnology). The membrane was rinsed with TBS-T and incubated with a horseradish-peroxidase-conjugated secondary antibody (Cat. No. sc-516102, 1:1000; Santa Cruz Biotechnology) for 2 h at room temperature. Finally, the membrane was detected using an ECL kit (Pierce Biotechnology).

### 4.7. Real-Time Quantitative Polymerase Chain Reaction (RT-qPCR)

After 4 or 7 days of culturing, total RNA was isolated using a TRIzol reagent (Thermo scientific Co., Waltham, MA, USA) according to the manufacturer’s instructions. cDNA was synthesized using a cDNA synthesis kit (ET21025, PhileKorea, Seoul, Korea) according to the manufacturer’s instructions. RT-qPCR was performed using a Magnetic Induction Cycler with a QuantiSpeed SYBR kit (QS105-05, PhileKorea, Seoul, Korea). The sequences of primers are listed in Table 1. Cycling conditions were as follows: initial denaturation at 95 °C for 5 °min, followed by 40 cycles of 90 °C for 30 °s, 60 °C for 40 s, and 72 °C for 40 s. The relative quantification of RNA was calculated using the 2^-ΔΔCT^ method. β-actin was amplified as an internal control.

### 4.8. Immunofluorescence Staining for β-Catenin Nuclear Translocation

Cells were grown on glass coverslips and incubated with PIISVYWK and FSVVPSPK for 4 days. The cells were rinsed with ice-cold PBS twice and fixed with 3.7% paraformaldehyde in PBS for 15 min, followed by permeabilization with 0.1% Triton X-100 in PBS for 15 min. The cells were blocked with 2% bovine serum albumin for 60 min and then incubated with a β-catenin antibody conjugated with Alexa Flour 488 (1:500 dilution, Santa Cruz Biotechnology) in 0.1% BSA for 3 h at room temperature, followed by staining with Hoechst 33342 (2 μg/mL) for 15 min. The cells were washed with PBS three times and viewed on a fluorescence microscope (Leica, Wetzlar, Germany).

### 4.9. Statistical Analysis

The data were analyzed using Sigma Plot 12.0 (Systat Software Inc., San Jose, CA, USA) and presented as mean ± standard deviation (S.D). Statistical analysis was performed using one-way analysis of variance. Values of * *p* < 0.05 and ** p < 0.01, or ^#^ *p* < 0.05 and ^##^ *p* < 0.01, were considered statistically significant.

## 5. Conclusions

In conclusion, this study demonstrated that blue mussel-derived antioxidant peptides PIISVYWK and FSVVPSPK promoted osteoblast differentiation by increasing ALP activity and the level of mineralization in hBMMSCs. The two peptides also increased osteoblastic differentiation markers, such as Runx2, OSX, and COL1A1. The mechanistic study revealed that the two peptides increased Wnt molecule signaling by transcriptional and translational regulation. In addition, pharmacological approaches suggested that the two peptide-mediated Wnt/β-catenin signaling pathway was involved in osteoblast differentiation of hBMMSCs. These findings may be helpful for new insights into peptide-mediated osteogenesis and developing peptide-based therapeutic agents for bone-related diseases.

## Figures and Tables

**Table 1:** RT-qPCR primers.

Genes	Forward Primer	Reverse Primer
*Wnt1*	AACAGCGGCGTCTGATAC	GCGGAGGTGATAGCGAAG
*Wnt3a*	ATGAACCGCCACAACAAC	TTCTCCACCACCATCTCC
*Runx2*	GGAGTGGACGAGGCAAGAGTTT	AGCTTCTGTCTGTGCCTTCTGG
*Osterix*	CCTCTGCGGGACTCAACAAC	AGCCCATTAGTGCTTGTAAAGG
*β-catenin*	AGCTTCCAGACACGCTATCAT	ATGACCCTGTAGGCAGAAACC
*COL1A1*	GACGTCCTGGTGAAGTTGGT	ACCAGGGAAGCCTCTCTCTC
*Cyclin D1*	CCCTCGGTCTCCTACTTCA	GTTTTCTCCTCCGCCTCT
*β-actin*	CAATGTGGCCGAGGACTTT	CATTCTCCTTAGAGAGAAGTGG
